# *Mclk1*^+/- ^mice are not resistant to the development of atherosclerosis

**DOI:** 10.1186/1476-511X-8-16

**Published:** 2009-05-05

**Authors:** Bryan G Hughes, Siegfried Hekimi

**Affiliations:** 1 Department of Biology, McGill University, Montreal H3A 1B1, Canada

## Abstract

**Background:**

Mice with a single copy of *Mclk1 *(a.k.a. *Coq7*), a gene that encodes a mitochondrial enzyme required for the biosynthesis of ubiquinone and other functions, live longer than wild-type mice. The prolonged survival implies a decreased mortality from age-dependent lethal pathologies. Atherosclerosis is one of the main age-dependent pathologies in humans and can be modeled in mice that lack Apolipoprotein E (*ApoE*^-/-^) or mice that lack the Low Density Lipoprotein Receptor (*LDLr*^-/-^) in addition to being fed an atherosclerosis-inducing diet. We sought to determine if *Mclk1 *heterozygosity protects against atherosclerosis and dyslipidemia in these models.

**Results:**

We found that *Mclk1 *heterozygosity did not protect against dyslipidemia, oxidative stress, or atherosclerosis in young (6 or 10 months) or older (18 months) mice. Furthermore, the absence of *ApoE *suppressed the lifespan-promoting effects of *Mclk1 *heterozygosity.

**Conclusion:**

These findings indicate that although *Mclk1 *heterozygosity can extend lifespan of mice, it does not necessarily protect against atherosclerosis. Moreover, in the presence of hyperlipidemia and chronic inflammation, *Mclk1 *heterozygosity is incapable of extending lifespan.

## Introduction

Median lifespan is increased by mutations in the gene *clk-1*, and by heterozygosity for a null allele of the mammalian *clk-1 *homologue *Mclk1*, in *Caenorhabditis elegans *and mice, respectively [1-3]. CLK-1/MCLK1 is a mitochondrial hydroxylase required for the biosynthesis of ubiquinone (UQ or Coenzyme Q) [4,5], a lipid-like molecule that plays a vital role as an electron transporter in the mitochondrial electron transport chain, is an important lipid-soluble antioxidant, and also performs a variety of other functions [6]. *Mclk1*-null (*Mclk1*^-/-^) cells and embryos make no UQ and the embryos are not viable, while *Mclk1 *heterozygous (*Mclk1*^+/-^) cells and mice are superficially normal and show normal levels of UQ [7,8]. While the biochemical phenotypes associated with *Mclk1 *heterozygosity have begun to be uncovered [3,9], the actual effect on the mouse in terms of resistance to the physical decline associated with age and to age-dependent disease has not yet been examined.

The longest-lived humans display an increased resistance to the common age-dependent diseases (cardiovascular disease, stroke, cancer, etc.), as the majority of them either develop these diseases later than the general population or not at all [10,11]. It is therefore tempting to expect a similar resistance to the development of age-dependent diseases in *Mclk1*^+/- ^and other lines of long-lived mice. One particularly prevalent age-dependent disease is atherosclerosis, in which the oxidative modification of low-density lipoprotein and the resultant recognition by cells of the immune system results in a chronic inflammatory state within the wall of blood vessels. This leads to the formation of a plaque containing a mixture of extracellular cholesterol deposits, immune cells and infiltrating smooth muscle cells [12]. Interestingly, manipulations that extend lifespan in mice have been shown to protect against atherosclerosis. Among long-lived mice, calorically restricted mice as well as genetically modified p66^shc-/- ^and UCP1 transgenic mice have been evaluated for atherosclerosis susceptibility. The fact that both were found to have an increased resistance to the disease [13-15] suggests that *Mclk1*^+/- ^mice may also be resistant to the disease.

A further incentive for studying the effect of *Mclk1 *heterozygosity on atherosclerosis is the finding that *clk-1 *worms have an altered lipid metabolism, consistent with a decrease in oxidatively modified lipoproteins [16]. Furthermore, the core non-lipid component of these particles appears to be vitellogenin, a protein that is homologous to apolipoprotein B, the main structural component of atherosclerosis-inducing lipoprotein particles in mammals [17,18]. The conservation of lipid metabolism between worms and mammals was further confirmed by the finding that several lipid-lowering drugs developed in mammals are active on lipoprotein metabolism in worms [19].

Atherosclerosis does not appear to develop in mice fed a regular diet, due to the partitioning of most cholesterol into the anti-atherogenic High Density Lipoprotein fraction of lipoproteins. Therefore, the lifespan extending effect of *Mclk1 *heterozygosity cannot be due to a decrease in atherosclerosis-related mortality. However, the underlying conditions thought to be responsible for atherosclerosis, oxidative stress and inflammation, are known to be strongly associated with aging, justifying the study of atherosclerosis in aging mice [20,21]. Mice lacking the Low Density Lipoprotein Receptor and fed a high-fat, high-cholesterol "Western" diet (*LDLr*^-/-^), as well as mice lacking apolipoprotein E fed a regular chow diet (*ApoE*^-/-^), develop hyperlipidemia and extensive aortic atherosclerosis, and are commonly used to model atherosclerosis in mice [22,23]. Although the lipid profile of these mice is characteristic of only the more dyslipidemic humans, the histopathological characteristics of the lesions appear similar to those observed in humans and other models, even if they develop over a shorter timeframe [24]. Furthermore, although coronary artery disease predominately affects those of middle-age or older, the atherosclerotic lesions that underlie the disease (and which are the focus of this study) begin to develop much earlier in life, with young adults having comparable aortic atherosclerosis to that observed in mice at an equivalent stage in their lives [25].

We elected to use two separate models because, although they theoretically affect the same system (the uptake of lipid from the circulation), they are known to respond differently to certain interventions [26-28]. Importantly, the *LDLr*^-/- ^model develops more severe atherosclerosis than the *ApoE*^-/- ^model, allowing us to measure the effect of *Mclk1 *heterozygosity on atherosclerosis of both medium and high severity. We also measured atherosclerosis in mice of different ages, in order to detect any possible impact of an age-dependent effect of *Mclk1 *heterozygosity on atherosclerosis. Here, we demonstrate that *Mclk1 *heterozygosity does not consistently affect atherosclerosis or lipid profile in either model. Furthermore, *Mclk1 *heterozygosity fails to extend lifespan in atherosclerosis-susceptible *ApoE*^-/- ^mice.

## Methods

### Animals and Diet

*LDLr*^-/- ^[29] and *ApoE*^-\- ^[30] mice on a C57Bl/6J background were purchased from The Jackson Laboratory (Bar Harbor, ME) and were crossed to *Mclk1*^+/- ^mice, previously produced by gene targeting [7], to produce *LDLr*^-/-^;*Mclk1*^+/+^, *LDLr*^-/-^;*Mclk1*^+/-^, *ApoE*^-\-^; *Mclk1*^+\+ ^and *ApoE*^-\-^; *Mclk1*^+\- ^animals. Genotypes were determined by PCR. Mice were maintained on 4.5% fat rodent chow (Charles River diet 5075). At 3 months of age mice in the *LDLr*^-/- ^groups were placed on a "Western"-type diet (Harlan Teklad, Madison, WI, TD.01444) containing 21% (w/w) anhydrous milk fat and 0.15% cholesterol. Mice in the *ApoE*^-/- ^groups were fed regular rodent chow throughout the experiments. Mice were housed in a specific pathogen free facility at McGill University, 2–5 animals per cage, and fasted overnight prior to sacrifice by anesthetic overdose (Ketamine/Xylazine/Acepromazine).

*ApoE*^-/- ^mice were sacrificed at 10 and 18 months of age and *LDLr*^-/- ^mice at 6 and 10 months of age (Table 1). 18 months was judged the greatest age that we could obtain without significant losses due to mortality in the *ApoE*^-/- ^group. We had originally intended to sacrifice the *LDLr*^-/- ^mice at 18 months as well, but we found that after 10 months of age on the "Western" diet *LDLr*^-/- ^mice developed severe health problems. The earlier measurement point for each model is the youngest age where we could reliably obtain measurable atherosclerosis in each case. For both groups, male and female mice were sacrificed at the earlier time-point, and females only at the later time-point.

**Table 1:** Experimental Design

Model	Age (months)	Gender(s)	Background (# Backcrosses)
*ApoE*^-/-^	10	M, F	CBA × C57BL/6J (4)
	10	M, F	129Sv/Balb/c × C57BL/6J (10)
	18	F	CBA × C57BL/6J (3)
	Lifespan	M, F	129Sv/Balb/c × C57BL/6J (6)
			
*LDLr*^-/-^	6	M, F	CBA × C57BL/6J (3)
	10	F	129Sv/Balb/c × C57BL/6J (6)

The genetic backgrounds of the mice varied somewhat between studies, although they were always consistent within a study (Table 1). Mice on the CBA background were backcrossed 3 times into the C57Bl/6J background to create the *LDLr*^-/- ^mice sacrificed at 6 months of age and mice on a mixed 129Sv × Balb/c background were backcrossed 6 times for *LDLr*^-/- ^mice sacrificed at 10 months of age. *ApoE*^-/- ^mice sacrificed at 10 and 18 months were backcrossed 4 and 3 times respectively out of the CBA background, and those used in the aging experiment were backcrossed 6 times out of a mixed 129Sv × Balb/c background. A second group of *ApoE*^-/- ^mice sacrificed at 10 months of age was backcrossed 10 times from the same background. In all cases, the C57BL/6J strain made the greatest contribution to the background, which is important because of this strain's relative sensitivity to atherosclerosis development [31], as well as because the ability of *Mclk1 *heterozygosity to extend lifespan in this strain has been confirmed in our lab [32].

To determine the lifespan of *Mclk1*^+/- ^mice in the *ApoE*^-/- ^background, mice were kept until either natural death, or evidence of impending mortality (such as sudden drastic weight loss, lack of movement or a severely distended abdomen) necessitating euthanasia.

All studies were approved by the McGill Faculty of Science Animal Care Committee and conducted according to the guidelines of the Canadian Council on Animal Care.

### Atherosclerosis Severity

The surface area of atherosclerotic lesions was measured on the inner surface of the aorta, from the aortic origin to the iliac branch point, as has been previously described [33,34]. Quantification of staining on the acquired images was carried out using UTHSCSA ImageTool v3.0 (University of Texas Health Science Center in San Antonio, USA), and results were expressed as percentage surface area of the aorta occupied by Oil Red O-staining lesions. The innominate, common carotid and subclavian arteries were excluded from the analysis.

### Plasma Measurements

EDTA plasma was collected by cardiac puncture of anaesthetized mice, flash-frozen in liquid nitrogen and stored at -80°C. Kits for measuring plasma cholesterol were obtained from Wako Chemicals USA and those for triglycerides from Sigma-Aldrich. Lipid peroxidation, as quantified by the level of malondialdehyde (MDA), was measured with a Thiobarbituric Acid Reactive Substances (TBARS) Assay Kit from ZeptoMetrix Corporation. The TBARS method of measuring MDA has received considerable criticism for being an insufficiently representative measurement of lipid peroxidation [35-37]. Despite this, even some of the harshest critics concede that the assay frequently yields useful results supported by other better-validated assays, although it may be better thought of as a measurement of susceptibility to lipid peroxidation rather than the steady-state level of damage [37].

### Statistics

The non-parametric Mann-Whitney test was used to compare atherosclerosis surface area in prepared aortas. Otherwise, the unpaired two-tailed Student's t-test was used. Survival curves were compared using the Log-rank (Mantel-Cox) test. Two-way ANOVA with Bonferroni posttests was used for comparing body-weights collected over lifespan. Comparisons were always made between genotypes, within genders, using Prism 4.03 (GraphPad Software, Inc).

## Results

### *Mclk1 *heterozygosity does not decrease the severity of atherosclerosis

**Figure 1:**
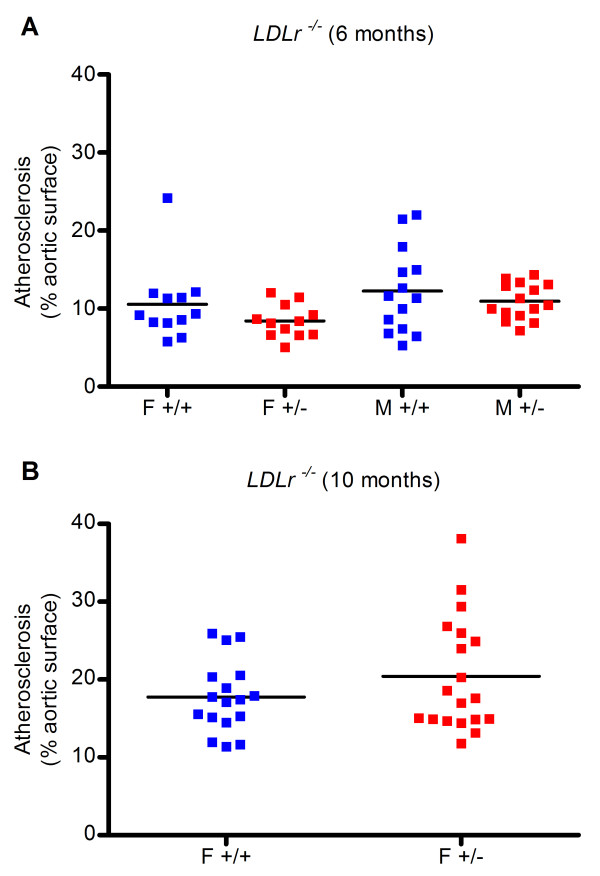
**Heterozygosity for *Mclk1 *does not prevent atherosclerosis in the *LDLr*^-/- ^disease model**. Atherosclerotic lesions that stain with the lipid-sensitive dye Oil Red O were quantified on the inner surface of the aorta in *LDLr*^-/- ^mice at (A) 6 months of age, with three backcrosses into C57BL/6J and (B) 10 months of age with six backcrosses into C57BL/6J. F and M labels stand for females and males, respectively. The +/+ and +/- labels stand for the *Mclk1*^+/+ ^and *Mclk1*^+/- ^genotypes, respectively.

**Figure 2:**
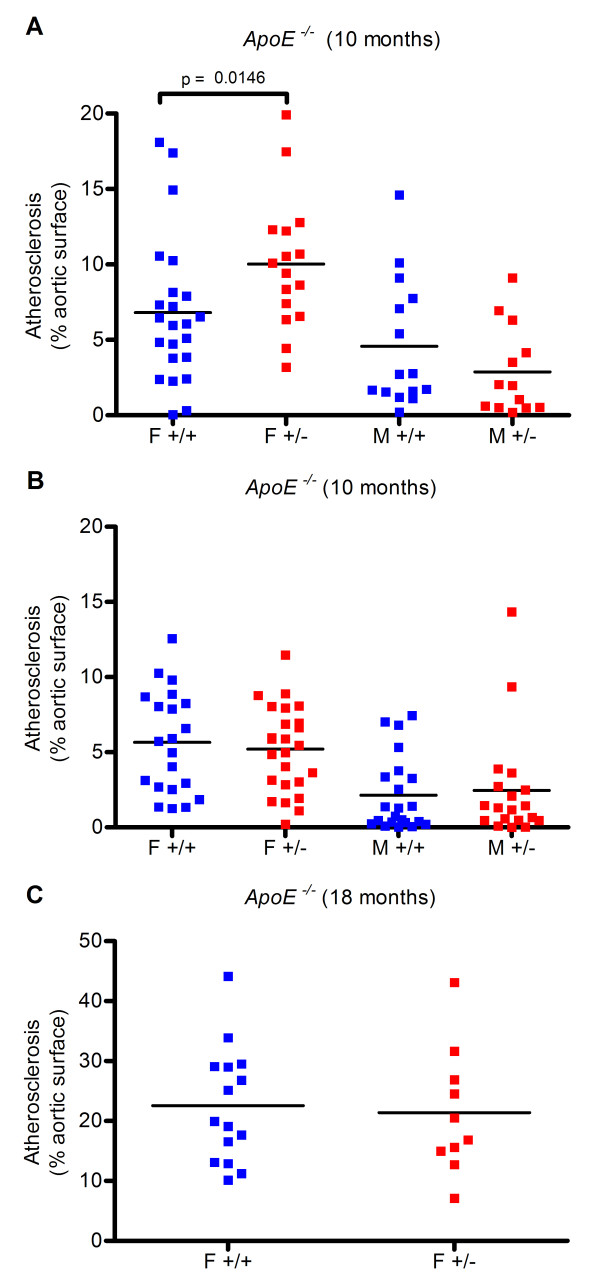
**Heterozygosity for *Mclk1 *does not prevent atherosclerosis in the *ApoE*^-/- ^disease model**. Atherosclerotic lesions that stain with the lipid-sensitive dye Oil Red O were quantified on the inner surface of the aorta in *ApoE*^-/- ^mice at (A) 10 months, initial trial with four backcrosses into C57BL/6J, (B) 10 months, second trial with ten backcrosses into C57BL/6J and (C) 18 months, with three backcrosses into C57BL/6J. F and M labels stand for females and males, respectively. The +/+ and +/- labels stand for the *Mclk1*^+/+ ^and *Mclk1*^+/- ^genotypes, respectively.

To determine if *Mclk1 *heterozygosity protected against atherosclerosis, *Mclk1*^+/- ^mice were crossed into atherosclerosis-sensitive *ApoE*^-/- ^and *LDLr*^-/- ^backgrounds. The proportion of the inner aortic surface occupied by atherosclerotic lesions was then quantified. Atherosclerosis in both the *ApoE*^-/- ^and *LDLr*^-/- ^models of the disease was not inhibited by *Mclk1 *heterozygosity (Figures 1 and 2). In one condition (*ApoE*^-/- ^mice sacrificed at 10 months of age), *Mclk1*^+/- ^females had increased atherosclerosis (6.8 vs. 10 percent of aortic surface area, p = 0.0146). In the same experiment, there was a 37 percent decrease in atherosclerosis in *Mclk1*^+/- ^males that was not statistically significant (p = 0.21). As the mice in this cohort had only been backcrossed for four generations, it is possible that high variability due to genetic heterogeneity could have been masking an effect of *Mclk1 *heterozygosity in males. We therefore repeated this study in a second group of *ApoE*^-/- ^mice that had been backcrossed ten generations onto the C57BL/6J background. In this second cohort of mice, there was no effect of *Mclk1 *heterozygosity in either gender.

In addition, *Mclk1 *heterozygosity in these atherosclerosis susceptible backgrounds did not produce any consistent change in lipid profiles (Table 2), including total plasma cholesterol and triglycerides. *Mclk1*^+/-^; *ApoE*^-/- ^males sacrificed at 10 months of age had decreased plasma triglycerides. However, when this experiment was repeated in the fully congenic background, triglycerides were not affected. Instead, we saw an increase in cholesterol levels. Plasma lipid peroxidation was never significantly affected. Body weight of female *LDLr*^-/-^; *Mclk1*^+/- ^mice sacrificed at six months of age was slightly decreased, but we did not see an effect on body weight in any other group.

**Table 2:** Plasma lipid characteristics and body weight at sacrifice (expressed as mean ± standard deviation)

	Age	Sex	*Mclk1*	Cholesterol (mg/dl)	Triglycerides (mg/dl)	TBARS (nmol MDA/ml)	Weight (g)
*ApoE*^-/-^	10 months (1)	F	+/+	501 ± 120	89 ± 28	33 ± 23	23.7 ± 2.8
			+/-	540 ± 97	109 ± 45	38 ± 12	25.4 ± 3.7
		M	+/+	529 ± 199	162 ± 51 *	42 ± 33	32 ± 3.5
			+/-	454 ± 166	118 ± 47*	23 ± 16	31.2 ± 2.3
	10 months (2)	F	+/+	337 ± 78	67 ± 20	8.6 ± 2.4	21.9 ± 1.5
			+/-	362 ± 67	63 ± 18	7.9 ± 2.4	21.6 ± 1.5
		M	+/+	267 ± 94 *	82 ± 27	9.4 ± 4.9	28.2 ± 1.8
			+/-	351 ± 112 *	83 ± 22	10.2 ± 6.3	27.9 ± 2.1
	18 months	F	+/+	726 ± 203	56 ± 24	24 ± 11	26.6 ± 3.4
			+/-	747 ± 151	67 ± 16	25 ± 12	28 ± 3.1
							
*LDLr*^-/-^	6 months	F	+/+	1599 ± 291	287 ± 130	58 ± 12	28.3 ± 4.8 *
			+/-	1600 ± 442	289 ± 119	51 ± 21	24.7 ± 3.4 *
		M	+/+	1700 ± 427	477 ± 184	75 ± 25	35.8 ± 4.5
			+/-	1723 ± 446	526 ± 223	76 ± 27	35 ± 4.2
	10 months	F	+/+	1390 ± 387	267 ± 98	24.4 ± 7.1	28.6 ± 4.2
			+/-	1343 ± 479	225 ± 71	20.8 ± 5.7	28.4 ± 3.4

*: p < 0.05

### Loss of *ApoE *suppresses the lifespan extension conferred by *Mclk1 *heterozygosity

**Figure 3:**
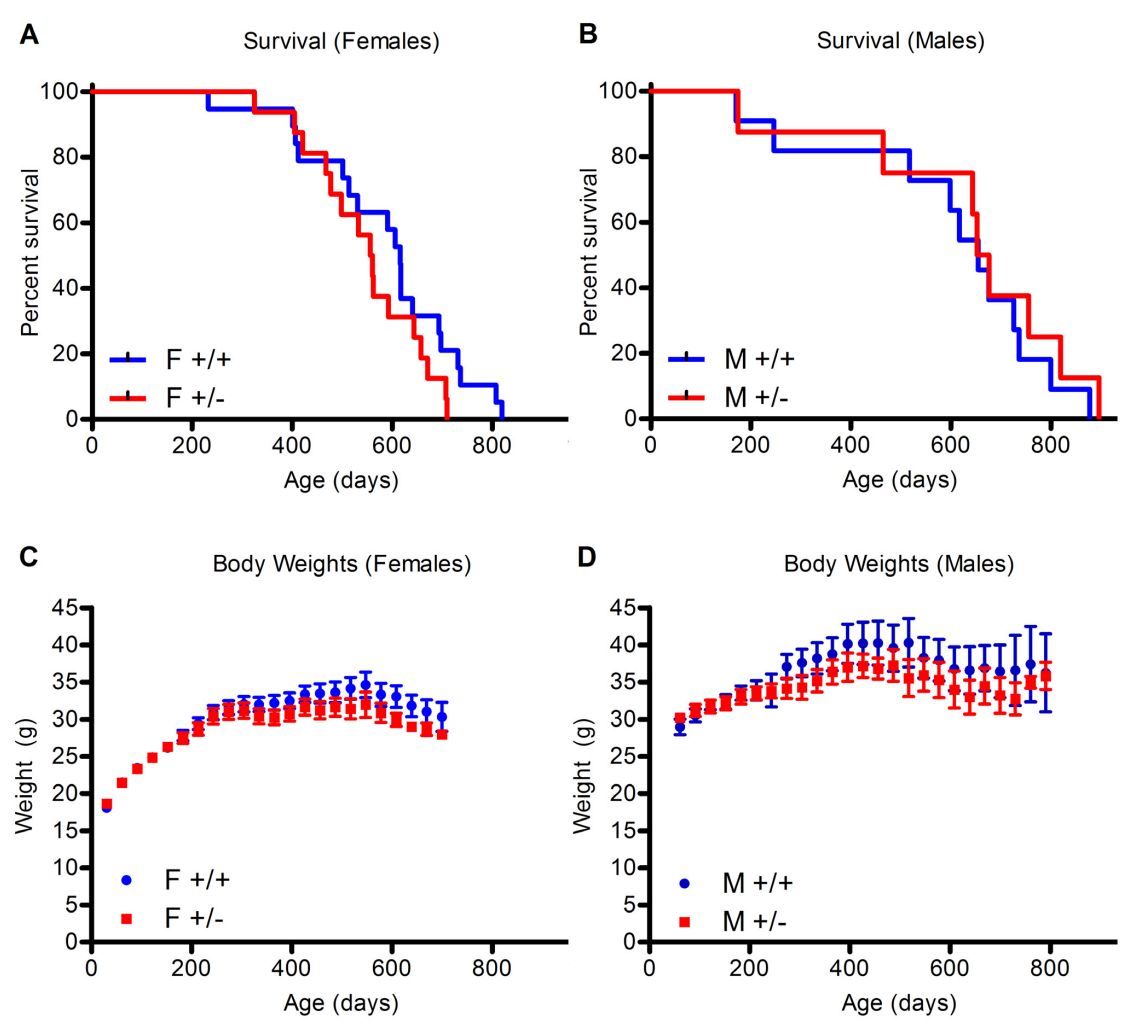
**Survival curves and body weights throughout lifespan of *Mclk1*^+/+ ^and^+/- ^mice on the *ApoE*^-/- ^background**. Survival of (A) female and (B) males was not affected by *Mclk1 *heterozygosity. Mice were weighed weekly, and the monthly average was plotted with the standard error of the mean for (C) females and (D) males. For both (C) and (D) the difference between genotypes was significant at p < 0.01. F and M labels stand for females and males, respectively. The +/+ and +/- labels stand for the *Mclk1*^+/+ ^and *Mclk1*^+/- ^genotypes, respectively.

Male and female *Mclk1*^+/- ^mice on an *ApoE*-null background failed to live longer than *Mclk1*^+/+ ^controls (Figure 3). Female *Mclk1*^+/- ^mice actually had a shorter average lifespan than controls, although this difference did not reach statistical significance (p = 0.16). In *Mclk1*^+/- ^mice of both genders, body weight was decreased throughout life (p < 0.0001 females, p = 0.0064 males) (Figure 3). 21 out of 35 females and 9 out of 18 males were euthanized as they were obviously near death due to severe illness (proportions were equal for each genotype), with the remainder dying naturally. Atherosclerosis was quantified in euthanized mice, and *Mclk1*^+/- ^females appeared to have decreased atherosclerosis relative to controls, although this difference did not reach statistical significance (16.4 ± 12.5 in *Mclk1*^+/+ ^vs. 9.6 ± 5.8 percent in *Mclk1*^+/-^, p = 0.18 by Mann-Whitney test). Atherosclerosis was not affected in males (20.3 ± 14.25 in wild-type vs. 23 ± 16.57 in *Mclk1*^+/-^, p = 0.63). In the group of *ApoE*^-/- ^mice sacrificed at 18 months of age, three mice of each genotype died prior to their date of sacrifice, closely paralleling the survival curve of mice in the lifespan study.

## Discussion

### The effect of *Mclk1 *heterozygosity on atherosclerosis

In five separate studies, we showed that *Mclk1 *heterozygosity did not ameliorate atherosclerosis in mice. In one study, female *Mclk1*^+/- ^mice showed a statistically significant increase in atherosclerosis, and males showed a non-significant decrease. However, we were unable to repeat either of these results in a follow-up study in which the mice were in a fully congenic background. Our experiments covered a range of ages and severity of disease, indicating that *Mclk1 *heterozygosity did not have any beneficial age-dependent or -independent effects on atherosclerosis. In other words, we only observed an effect on atherosclerosis in one particular genetic background, suggesting that it was due to a unique set of interactions within this particular background. This forces us to conclude that any potential effects of *Mclk1 *heterozygosity on atherosclerosis would not be due to the same mechanism as that which produces lifespan extension, which was observed in several separate backgrounds.

We have recently reported that young *Mclk1*^+/- ^mice have decreased lipid peroxidation, measured via plasma isoprostanes. At the same time, the mice had substantial alterations in mitochondrial function, including a general decrease in mitochondrial respiration and an increase in mitochondrial oxidative stress [9]. Mitochondrial dysfunction linked to increased oxidative stress, such as that found in *Mclk1*^+/- ^mice, has been shown to increase susceptibility to atherosclerosis [38]. The balance between mitochondrial, atherosclerosis-sensitizing and systemic, potentially atherosclerosis-protective, phenotypes of *Mclk1*^+/- ^mice may explain the general absence of a significant effect on atherosclerosis that we observed. This balance may be modulated by genetic background, which could account for some of the contrasting results we observed between *ApoE*^-/- ^mice of different backgrounds. Furthermore, *Mclk1*^+/- ^mice also have higher levels of several pro-inflammatory cytokines (unpublished data). Inflammation plays a role in all stages of atherosclerosis [39], suggesting another possible reason why *Mclk1*^+/- ^mice are not protected against the disease.

### The effect of mutant *clk-1 *in *C. elegans *and *Mclk1 *in mice on lipid metabolism

*clk-1 *mutant worms have altered oxidative and lipid metabolism [16,19]. The organismal differences between worms and mice, as well as other differences between the two models (homozygous mutants in worms and heterozygous mutants in mice) may explain why we did not the see the expected effect on lipid metabolism and atherosclerosis in *Mclk1*^+/- ^mice. The *clk-1 *mutant worms are homozygous for alleles incapable of producing UQ, resulting in an obvious, easily observable, phenotype [2,40]. On the other hand, mice retain one copy of the wild-type allele, and the MCLK1 produced appears sufficient for a normal level of UQ synthesis [3,7,9]. It appears that, in both worms and mice, *MCLK1*/*CLK-1 *has an additional function besides the production of UQ. This is supported by evidence from both organisms. In worms, two mutant alleles of *clk-1 *yield phenotypes of different severity, despite the fact that neither allele can produce UQ [41,42]. *Mclk1*^+/- ^mice actually have several very clear biochemical phenotypes despite wild-type levels of UQ [3,9]. Yet, the absence of a lipid phenotype in mice suggests the existence of a threshold that is not reached when CLK-1/MCLK1 activity is only reduced as in *Mclk1*^+/- ^mouse mutants rather than completely abolished or very severely reduced as in the *C. elegans *mutants.

### Suppression of *Mclk1*^+/- ^longevity by loss of *ApoE*

In mice lacking the ApoE protein, *Mclk1 *heterozygosity no longer extends lifespan. In other words, some characteristic of the *ApoE*^-/- ^background actually shortens the lifespan of *Mclk1*^+/- ^mice relative to *Mclk1*^+/+ ^mice. We also observed that *Mclk1*^+/-^; *ApoE*^-/- ^mice weighed slightly less than *Mclk1*^+/+^; *ApoE*^-/- ^mice, starting at approximately 1 year of age. This is in contrast to older *Mclk1*^+/- ^mice wild-type for *ApoE*, which either weigh the same or more than wild-type controls [unpublished data and [1]]. This does imply that the absence of *ApoE *has detrimental effects on *Mclk1*^+/- ^mice. An increased prevalence of atherosclerosis is unlikely to be the cause of this lifespan shortening effect. Firstly, as described above, *Mclk1*^+/- ^mice do not appear more susceptible to atherosclerosis development. If anything, *Mclk1*^+/- ^female mice in the aging experiment that were euthanized due to impending death showed a trend of slightly less aortic atherosclerosis than their wild-type controls. Secondly, it has been surprisingly difficult to find evidence in *ApoE*^-/- ^mice of the arterial occlusion-induced cardiovascular events that are so lethal to humans [43]. Although older *ApoE*^-/- ^mice may develop atherosclerosis of the coronary arteries, only a small proportion of these are found to display evidence of more advanced coronary artery disease, such as plaque rupture [24,44]. Aside from atherosclerosis, *ApoE*^-/- ^mice suffer from other conditions, such as increased oxidative stress [45] and inflammation [46]. As described above, young *Mclk1*^+/- ^mice have higher levels of oxidative stress in mitochondria as well as greater expression of certain cytokines consistent with a more pro-inflammatory state. Although these factors by themselves clearly do not prevent the increased longevity of *Mclk1*^+/- ^mice, they may exert a negative effect that cancels out the lifespan extension when combined with corresponding phenotypes in *ApoE*^-/- ^mice.

## Conclusion

Contrary to our expectations based on the increased longevity of *Mclk1*^+/- ^mice, *Mclk1 *heterozygosity does not protect against atherosclerosis. This is not surprising in light of recent findings that suggest a complex pattern of phenotypes due to *Mclk1 *heterozygosity, some of which may actually facilitate development of a disease such as atherosclerosis that involves oxidative stress and inflammation. Furthermore, *Mclk1 *heterozygosity does not extend lifespan in the *ApoE*-null background, suggesting that *Mclk1 *heterozygosity is not capable of protecting against the increased oxidative stress and inflammation that afflict *ApoE*-null mice.

## Competing interests

The authors declare that they have no competing interests.

## Authors' contributions

BH participated in the conception and design of this study, performed the experiments, participated in the analysis of data, and helped write the paper. SH participated in the conception and design of the study, participated in the analysis of data, and helped write the paper.

## Authors' Information

SH is Campbell Chair of Developmental Biology and Strathcona Chair of Zoology.
